# Supplementary material for: Cell-wall synthesis and ribosome maturation are co-regulated by an RNA switch in Mycobacterium tuberculosis
Source: Nucleic Acids Res. 2018 Mar 29;46(11):5837–49. doi: 10.1093/nar/gky226 (PMC6009663; doi:10.1093/nar/gky226)
Supplement: Supplementary Data [file gky226_supplemental_files.pdf]

Supplementary Materials

Table S1: plasmids

Construct	Source	Oligonucleotides
pGEM	pGEM T-easy II (Promega)	-
pCR4 TOPO	TOPO TA Cloning Kit with PCR4 TOPO (Invitrogen)	-
pKA425	(1)	-
pKA425p	pKA425 with PCLI heterologous promoter (1) inserted between XbaI and HindIII	3.64 and 3.65
425L-RpfB-ATG	pKA425 translational fusion of RpfB from -355 bp upstream of and including the annotated ATG start codon in fusion with *lacZ*	2.20 and 2.34
425L-TLSM-2	425L-RpfB-ATG with single basepair deletion +151 bp downstream of the P1 TSS	5.08 and 5.09
425L-TLSM-1	425L-RpfB-ATG with single basepair deletion +191 bp downstream of the P1 TSS	2.49 and 2.50
425L-TLSM+1	425L-RpfB-ATG with double basepair deletion in *lacZ* CDS at +139 to +140 bp downstream of the ATG start codon	2.64 and 2.65
425L-TLSM-GTc	425L-RpfB-ATG with GTG to GTc substitution +143 bp downstream of the P1 TSS	5.17 with 2.40 and 5.16 with 2.73
425L-TLSM-TTa	425L-RpfB-ATG with TTG to TTa substitution +179 bp downstream of the P1 TSS. Disrupts TTG start codon	5.19 with 2.40 and 5.18 with 2.73
425L-TLSM-AaG	425L-RpfB-ATG with ATG to AaG substitution +217 bp downstream of the P1 TSS	2.70 and 2.71
425L-TLSM-RBS*	425L-RpfB-ATG with GAGGTCGGGGA to ctccTCcccct substitution +156 to +166 bp downstream of the P1 TSS	2.74 and 2.75
425L-RpfB-RBSWterm	425L-RpfB-ATG with G to C substitution +112 bp downstream of the P1 TSS	2.79 and 2.80
425L-RpfB-RBSWanti	425L-RpfB-ATG with U to C substitution +6 bp downstream of the P1 TSS	5.02 and 5.03
425L-RpfB-RBSWdel	425L-RpfB-ATG with deletion spanning +1 bp to +127 bp of the P1 TSS	5.37 and 5.38
425L-RpfB-RBSWpolA	425L-RpfB-ATG with U to A substitutions at +117 to +119 bp downstream of the P1 TSS	5.33 with 2.40 and 5.32 with 2.73
425L-RpfB-P1*P2	425L-RpfB-ATG with TAGGGT to cAGGGc substitution -12 to -7 bp upstream of the P1 TSS	8.37 with 2.40 and 8.36 with 2.73
425L-RpfB-P1P2*	425L-RpfB-ATG with TACCGT to cACCGc substitution +68 to +73 bp downstream of the P1 TSS	8.39 with 2.40 and 8.38 with 2.73
425L-RpfB-P1*P2*	425L-RpfB-P1*P2 with TACCGT to cACCGc substitution +68 to +73 bp downstream of the P1 TSS	8.39 with 2.40 and 8.38 with 2.73
425S-RpfB-asRNA100	pKA425 transcriptional fusion of RpfB asRNA spanning -100 bp upstream of the asRNA TSS to +2 bp	2.68 and 2.69

pIRATE-P1RpfB	pIRATE (1) construct rrnA promoter replaced with with translational fusion of RpfB from -355 bp upstream of and including the annotated ATG start codon in fusion with *lacZ* inserted between XbaI and NcoI	2.53 and 2.34
pIRATE-P1RpfB+asRNA	pIRATE-P1RpfB with 149 bp asRNA insert spanning +1 to +149 bp of the asRNA TSS inserted between SpeI and XhoI	5.11 and 5.12
pGAMrrnX_haltRBSW	pGEM containing *M. smegmatis* ribosomal rrnB promoter spanning -80 to -8 bp of the TSS (2), inclusive of extended 'TGN' -10 motif. GTAAA insertion downstream if rrnB TSS. Transcriptional fusion of +1 to +176 of the *rpfB* P1 TSS fused downstream of the GTAAA insertion. The entire template was inserted within T-A cloning site. Construction resulted in a 42 bp deletion of the pGEM backbone between the vector NdeI restriction site and T-A cloning site and was therefore renamed pGAM	5.59 and 5.60 (ext_rrnB)<br>5.15 and 5.23 (RBSW)<br>5.79 and 5.23 (halt)
pGAMrrnX_haltRBSWanti	pGAMrrnX_haltRBSW with U to C substitution at +11 bp downstream of rrnB TSS	8.03 and 5.23
pGAMrrnX_haltRBSWterm	pGAMrrnX_haltRBSW with G to C substitution at +117 bp downstream of rrnB TSS	5.79 and 5.23
pGAMrrnX_haltRBSWpolA	pGAMrrnX_haltRBSW with U to A substitutions at +122 to 124 bp downstream of rrnB TSS	5.79 and 5.23
pGAMrrnX_haltRBSW_STOP	pGAMrrnX_RBSW with *synB* synthetic terminator (3) inserted +186 bp downstream of the rrnB TSS	8.44 and 8.45
pGAMrrnX_haltRBSWanti_STOP	pGAMrrnX_haltRBSW_STOP with U to C substitution at +11 bp downstream of rrnB TSS	8.44 and 8.45
pGAMrrnX_haltRBSWterm_STOP	pGAMrrnX_haltRBSW_STOP with G to C substitution at +117 bp downstream of rrnB TSS	8.44 and 8.45
pGAMrrnX_haltRBSWpolA_STOP	pGAMrrnX_haltRBSW_STOP with U to A substitutions at +122 to 124 bp downstream of rrnB TSS	8.44 and 8.45

Table S2: oligos

Number	Sequence	Use
1.48	GTCCCATTCCGAACCCGGAAGCTAAGCCTGCCAGCGCCTGTCTC	Template for ribo-probe
2.07	GCTGTCAACGATACGCTACGTAACGGCATGACAGTGTTTTTTTTTTTTTTTTTTTTTTT	3'RACE
2.09	GCTGTCAACGATACGCTACGTAACGGC	3'RACE
2.15	ATTGGCCGGTTCGTTACC	3'RACE
2.20	GCAGTCGATCGTACGCTAGT	Cloning
2.34	ATATATCCATGGGTGATTGGGTCTGATGAA	Cloning
2.40	ACGAGGGGCATTCACACCAGATTG	Cloning
2.49	CAAACTTCATCAGACCCAATC	Cloning
2.50	TAAGTAGAGTCAACGCGC	Cloning
2.53	ATTATCTAGACCACCGGGGCTTGGCGAACGAA	Cloning
2.64	CGTTTTACAACGTCGTGACTGGGAA	Cloning
2.65	CGGGATCATCCATGGGTGATTG	Cloning

2.68	TAATAAGCTTCGCGTTGACTCTACTTACCAAAC	Cloning
2.69	ATTATCTAGACATAGCCACCGGCGAACG	Cloning
2.70	CCAATCACCCAAGGATGATCCCG	Cloning
2.71	GTCTGATGAAGTTTGGTAAGTAGAG	Cloning
2.73	GCAGTTTGAGGGGACGACGACAGTATC	Cloning
2.74	CCCCTTATAGCGCGTTGACTCTAC	Cloning
2.75	GAGGAGGGCAACACTCAACCACCT	Cloning
2.79	GTTTCGGAGGCCCCATTTTGCTTTTTG	Cloning
2.80	CTAGAGGCCCCACCCGTT	Cloning
3.64	CTAGACGCTGACCACCCCCAGAACCCGAAGTTGACTCAAGTTCATTGGACTTGGT ACAGTGA	Cloning
3.65	AGCTTCACTGTACCAAGTCCAATGAACTTGAGTCAACTTCGGGTTCTGGGGGTGG TCAGCGT	Cloning
5.02	GATGCGCCAACGAGCGCGCCG	Cloning
5.03	CACCCTAGCCCATAAGCTCGG	Cloning
5.08	TTGCCGAGGTCGGGGATATAGC	Cloning
5.09	ACTCAACCACCTACACAGCGAAC	Cloning
5.11	TAATACTAGTGCTATATCCCCGACCTCG	Cloning
5.12	ATTACTCGAGATAACACCCGCGCGCCGG	Cloning
5.15	ATTAACTAGTGCCAATGAGCGCGCCG	Cloning
5.16	GTTCGCTGTGTAGGTCGTTGAGTGTT	Cloning
5.17	AACACTCAACGACCTACACAGCGAAC	Cloning
5.18	GGGGATATAGCGCGTTAACTCTACTTACC	Cloning
5.19	GGTAAGTAGAGTTAACGCGCTATATCCCC	Cloning
5.22	CGCGCCGAGCGGCCCATAACACCCGCGCGCCGGAGTTGCTCCTGTCTC	Template for ribo-probe
5.23	ATTACTCGAGCGCGCTATATCCCCGACC	Cloning/IVT template
5.31	CATATGGGTGACCGCGTCTGA	Cloning/IVT template
5.32	GGCCCAAAATGCTTTTTGTTCGCTG	Cloning
5.33	CAGCGAACAAAAAGCATTTTGGGCC	Cloning
5.37	GTTCGCTGTGTAGGTGGTTGAGTGTTGC	Cloning
5.38	GCATCCACCCTAGCCCATAAGCTCG	Cloning
5.48	ATTAAAGCTTGAGGTCGGGGATATAGCGCGTTG	Cloning
5.49	TAATCCATGGCAACCACCCGTCGCACCGTG	Cloning
5.50	ATTAAAGCTTGTCAAGGTTGGGGCGCCTGG	Cloning
5.51	AACGGCGGGCTGCGGTATGC	Cloning
5.59	TATGGGTGACCGCGTCTGACCAGGGAAAATAGCCCTCTGACCTGGGGATTTGAC TCCCAGTTTCCAAGGTGGTAACTTA	Cloning
5.60	CTAGTAAGTTACCACCTTGGAAACTGGGAGTCAAATCCCCAGGTCAGAGGGCTAT TTTCCCTGGTCAGACGCGGTCACCCA	Cloning
5.79	ACTTACTAGTGTAAAGCCAATGAGCGCGCCGAGCGGCCCA	Cloning
8.01	ATTAAAGCTTCCTCGCCACCCGCGAAGAGC	Cloning
8.03	ACTTACTAGTGTAAAGCCAACGAGCGCGCCGAGCGGCCCA	Cloning
8.04	GTCTTGCGCCGCTGCGCGAA	qRT-PCR
8.06	CGCGCTATATCCCCGACCTCG	IVT template amplification

8.07	GGACCCAGGATGTGACGTTC	qRT-PCR
8.08	TGCACACCACCGTAATACCC	qRT-PCR
8.09	ATCTGCTTGTCGAGTTCCCG	qRT-PCR
8.10	AGGGCGAGGTCTCATATCGA	qRT-PCR
8.11	GAGCGGCCCATAACACCC	qRT-PCR
8.36	CTTATGGGCCAGGGCGGATGCGCCA	Cloning
8.37	TGGCGCATCCGCCCTGGCCCATAAG	Cloning
8.38	GCCGGTTCGTCACCGCCTTGTGATC	Cloning
8.39	GATCACAAGGCGGTGACGAACCGGC	Cloning
8.43	CCGCGTTGAAAAAAAAAAGCGCC	IVT template amplification
8.44	TCGAGCGCCGCAACTGCGGCGCTTTTTTTTTTCAACGCGGATCCTAATAATCG	Cloning
8.45	AATTCGATTATTAGGATCCGCGTTGAAAAAAAAAAGCGCCGCAGTTGCGGCGC	Cloning
8.53	CGTTCGATCACAAGACGGTA	qRT-PCR
8.56	TCAACATTGGCCGGTTCGTT	qRT-PCR
8.57	GACTACCAGGCGCAACATCG	qRT-PCR
8.58	TTATGGGCTAGGGTGGATGC	qRT-PCR
8.62	CGCCAGATAGAGGTTGACCTTTCCGGG	qRT-PCR

Supplementary methods

***In vitro* transcription**

Using *mfold* (4) we found that adding GUAAA to the 5' end of the RpfB 5' UTR did not alter the predicted structures. This enabled halting the RNA polymerase at position 11. Templates were generated by PCR amplification of the pGAMrrnX vector, agarose gel extraction of the amplicon which itself was then used as template in a second PCR. Amplified template was ethanol precipitated and re-suspended in DEPC treated H_2O (Ambion) at 200 nM. The 302 basepair transcription template contained a -80 to -8 *rrnB* promoter fragment from pKA303(5) in which the -10 box (acgTAACTT) was modified to an extended -10 box (TGnTAACTT) to enhance factor independent initiation. This was followed by a SpeI site and the sequence 'GTAAA' fused with the *rpfB* riboswitch sequence spanning from the P1 TSS +1 to +176 bp followed by the *synB* synthetic terminator for termination at 211 nt (3).

In vitro transcription reactions were carried out using *E. coli* RNA polymerase holoenzyme (NEB). Halted TECs were assembled first with the addition of 5x reaction buffer (40 mM Tris-HCl, 150 mM KCl, 10 mM $MgCl_2$, 1 mM DTT, 0.01% Triton X-100, pH 7.5), 20 nM dsDNA template, 40 U 'RNasin plus' RNAse inhibitor (Promega) and 34 nM *E. coli* holoenzyme in 22.5 µL with DEPC treated H_2O. Reactions were incubated 37°C for 5 min before adding 2.5 µL pre-warmed 10x halt mix (1500 µM GpU dinucleotide, 25 µM GTP/CTP with 7.5 µM ATP (Promega), and 0.4 µCi/µl 32P-αATP (3000 Ci/mmol, Perkin Elmer). Reactions were incubated at 37°C for 15 min. After incubation pre-warmed heparin (Sigma) was added to 200 ng/µL to prevent additional transcription initiation in 45 µL with 1x buffer, incubating at 37°C for 5 min. Transcription elongation was activated by the addition of 5 µL pre-warmed 10x elongation mix (500 µM of all rNTPs) to yield a final 1x concentration of 50 µM final in the total 50 µL reaction. A 10x elongation mix of 2500 µM rNTP was used for reactions where a final concentration of 250 µM rNTP was required in the total 50 µL reaction.

Reactions extended at 37°C for 20 min before being stopped with 50 µL 2x formamide stop buffer (95% (v/v) formamide, 18 mM EDTA, 0.025% (w/v) SDS and trace amounts of xylene cyanol and bromophenol blue). Time course reactions were extracted as indicated. Reactions were stored at -20°C prior to use. Where required, NusA (NEB) was added to reactions during incubation with heparin.

Transcription reactions were separated using denaturing 7 M urea PAGE 10% (v/v) 19:1 acrylamide:bis-acrylamide (MP Bio Science), exposed to a phosphorimager screen and developed using Typhoon FLA 9500 (GE), sizing transcripts using radiolabelled Decade and Century RNA markers (Ambion).

β-galactosidase promoter fusions

Constructs utilised for promoter fusions in Figure 1C correspond the following in Table S1:
425=vector; WT=425L-RpfB-ATG; P1*=425L-RpfB-P1*P2; P2*=425L-RpfB-P1P2*;
P1*P2*=425L-RpfB-P1*P2*; P_{as}=425S-RpfB-asRNA100.

β-galactosidase assay for translational start site mapping (TLSM)

Constructs utilised for TLSM in Figure 2 correspond to the following in Table S1:
425=vector; WT=425L-RpfB-ATG; GTG-ORF=425L-TLSM-2; TTG-ORF=425L-TLSM-1;
ATG-ORF=425L-TLSM+1; GTG>GTc=425L-TLSM-GTc; TTG>TTa=425L-TLSM-TTa;
ATG>AaG=425L-TLSM-AaG; SD mut=425L-TLSM-RBS*.

β-galactosidase assay for riboswitch reporter fusions

Constructs utilised for riboswitch reporter fusions in Figure 4 correspond the following in Table S1:
425=vector; WT=425L-RpfB-ATG; ΔRBSW=425L-RpfB-RBSWdel;
U6Cterm=425L-RpfB-RBSWterm; G112Canti=425L-RpfB-RBSWanti; U117-119A=425L-RpfB-RBSWpolA.

β-galactosidase assay for integrated reported and target expression (pIRATE)

Constructs utilised for assaying asRNA influence on riboswitch/RpfB expression in Figure S2 correspond to the following in Table S1:
WT=pIRATE-P1RpfB; WT+asRNA=pIRATE-P1RpfB+asRNA.

Alignment of the *tatD-rpfB* intergenic regions

The multiple alignment of tatD-rpfB intergenic regions was built in stages following the rationale that the “switch” part of the intergenic region would be aligned optimally using a structure-guided alignment whereas the rest of the intergenic region should be aligned

using a sequence-based alignment, aligning the most similar sequences first. Hence, the predicted "switch" regions of species with P1 promoters and predicted terminators (*M. tuberculosis* H37Rv (NC_000962.3); *M.canettii* CIPT 140010059 (HE572590.1); *M. canettii* CIPT 140070017 (FO203510.1); *M. canettii* CIPT 140070010 (FO203509.1); *M. marinum M* (NC_010612.1); *M. ulcerans subsp. shinshuense* (AP017624.1)) were aligned first using the software LocaRNA (6) available at: http://rna.informatik.uni-freiburg.de/LocARNA/. This alignment was then used as a seed to align the whole *tatD-rpfB* intergenic regions of the same set of species using the online server for MAFFT (version 7(7), available from: http://mafft.cbrc.jp/alignment/software/.

To this initial alignment, three more species were added: *M. shinjukuense* strain CCUG 53584 (NZ_MVIK01000013.1); *M. kansasii* strain 9MK (CP019888.1); *and M. avium 104* (CP000479.1) using the *20PAM* scoring matrix. Finally, intergenic regions from the more phylogenetically distant species: *M. leprae* TN (AL450380.1); *M.abscessus* (NC_010397.1); *M. smegmatis str. MC2 155* (CP000480.1) were added to the previous alignment using the *200PAM* scoring matrix. For all MAFFT alignments, the program *mafft-add* was used to retain the initial alignment whilst adding new sequences, in combination with the more accurate iterative refinement method *G-INS-i*.

When aligning intergenic regions, approximately 10 codons (30 nucleotides) were added on either end to anchor the alignment. The use of additional nucleotides beyond the annotated intergenic region for each species are required in this case because the promoter regions and putative "switch" in *M. tuberculosis* overlap the coding region of *tatD* and the annotated CDS for *rpfB* are not consistent across mycobacterial species. Moreover, the start of the *rpfB* coding region of *M. avium* is annotated as 1090233 but it is clear from alignment to the *M. tuberculosis rpfB* that this could be extended 22 codons further upstream, hence the intergenic region plus buffer that was used for our alignment ends at nucleotide 1090196 instead. The three additional species whose intergenic regions appear more diverged are: *Mycobacterium leprae* TN (AL450380.1:314669-315016); *Mycobacterium abscessus* (NC_010397.1:1141551-1141702); *Mycobacterium smegmatis str. MC2 155* (CP000480.1:c5523574-5523378). In *M. abscessus*, *tatD* appears to end prematurely compared with *tatD* from *M. tuberculosis*, hence the region used in the alignment did not include a *tatD* buffer "anchor" in this case.

MAFFT-produced alignments were saved in clustal format and reloaded into Jalview (8) to produce the relevant figures.

Prediction of terminators in intergenic regions

The program TransTermHP (v2.09) (9) was used to predict putative terminators in the intergenic regions between tatD and rpfB. Default parameter values were used for all searches (windows of DNA of length 6 containing at least 3 thymines were treated as potential terminator tails; each side of the putative stem had to be at least 4 nucleotides long; the length of the loop had to be between 3 and 13 nucleotides long; the total length of the stem+loop had to be less than 59 nucleotides). Putative terminators were returned if both the hairpin score and the tail score were less than the default cut-off values of -2 and -2.5 respectively. Where no terminators were predicted, the program *2ndscore* from the same software suite (TransTermHP) was used (with default parameters) to predict the best hairpins

starting at every nucleotide in the sequence. The best scoring, non-overlapping hairpins in the region of the *M.tuberculosis* predicted terminator were selected.

1. Moores, A., Riesco, A.B., Schwenk, S. and Arnvig, K.B. (2017) Expression, maturation and turnover of DrrS, an unusually stable, DosR regulated small RNA in Mycobacterium tuberculosis. *PloS one*, **12**, e0174079.
2. Arnvig, K.B., Gopal, B., Papavinasasundaram, K.G., Cox, R.A. and Colston, M.J. (2005) The mechanism of upstream activation in the rrnB operon of Mycobacterium smegmatis is different from the Escherichia coli paradigm. *Microbiology*, **151**, 467-473.
3. Czyz, A., Mooney, R.A., Iaconi, A. and Landick, R. (2014) Mycobacterial RNA polymerase requires a U-tract at intrinsic terminators and is aided by NusG at suboptimal terminators. *mBio*, **5**, e00931.
4. Zuker, M. (2003) Mfold web server for nucleic acid folding and hybridization prediction. *Nucleic acids research*, **31**, 3406-3415.
5. Arnvig, K.B., Comas, I., Thomson, N.R., Houghton, J., Boshoff, H.I., Croucher, N.J., Rose, G., Perkins, T.T., Parkhill, J., Dougan, G. *et al.* (2011) Sequence-based analysis uncovers an abundance of non-coding RNA in the total transcriptome of Mycobacterium tuberculosis. *PLoS Pathog*, **7**, e1002342.
6. Will, S., Joshi, T., Hofacker, I.L., Stadler, P.F. and Backofen, R. (2012) LocARNA-P: accurate boundary prediction and improved detection of structural RNAs. *RNA*, **18**, 900-914.
7. Katoh, K. and Standley, D.M. (2013) MAFFT multiple sequence alignment software version 7: improvements in performance and usability. *Mol Biol Evol*, **30**, 772-780.
8. Waterhouse, A.M., Procter, J.B., Martin, D.M., Clamp, M. and Barton, G.J. (2009) Jalview Version 2--a multiple sequence alignment editor and analysis workbench. *Bioinformatics*, **25**, 1189-1191.
9. Kingsford, C.L., Ayanbule, K. and Salzberg, S.L. (2007) Rapid, accurate, computational discovery of Rho-independent transcription terminators illuminates their relationship to DNA uptake. *Genome Biol*, **8**, R22.

Supplementary Figures

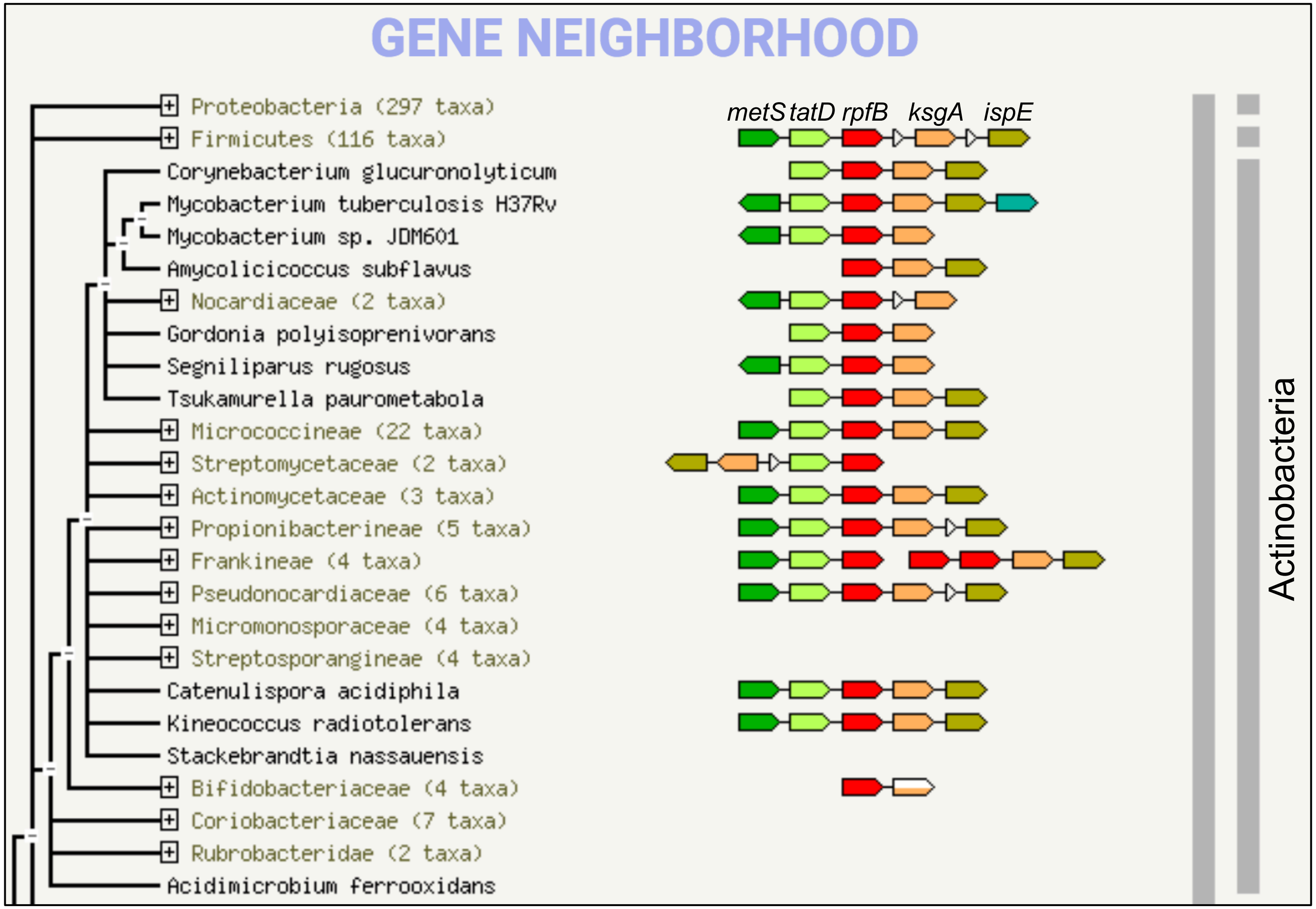

Figure S1: Conservation of the *rpfB* locus. The image shows the *rpfB* neighbourhood, according to STRING (https://string-db.org). The five genes, *metS, tatD, rpfB, ksgA* and *ispE* reside in the same locus in a range of species. Small, white arrows indicate non-conserved genes.

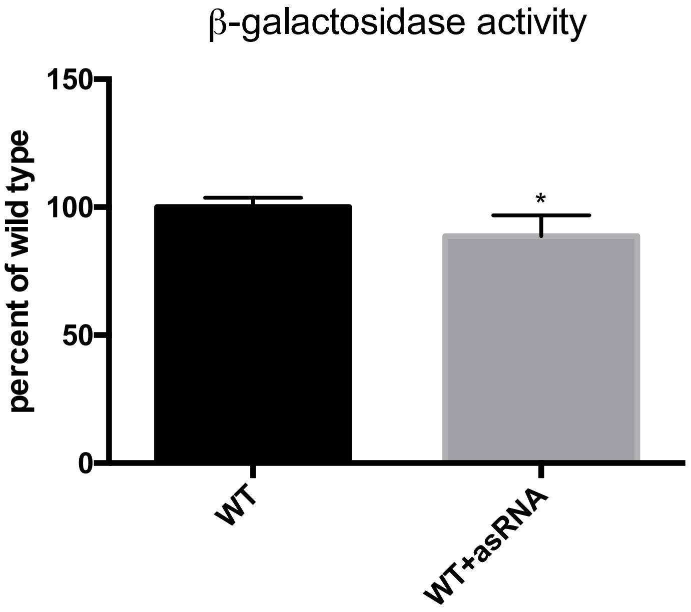

Fig. S2: β-gal activity of *rpfB-lacZ* reporter in the presence and absence of asRNA expression. The rpfB 5' UTR and first codons (from +1 to ATG at +215 in Fig. 1) was cloned between a constitutive promoter and *lacZ* in pIRATE-P1RpfB (Table S1). The asRNA was fused to another, divergently expressed heterologous promoter. Results indicate mean and SD of three biological replicates.

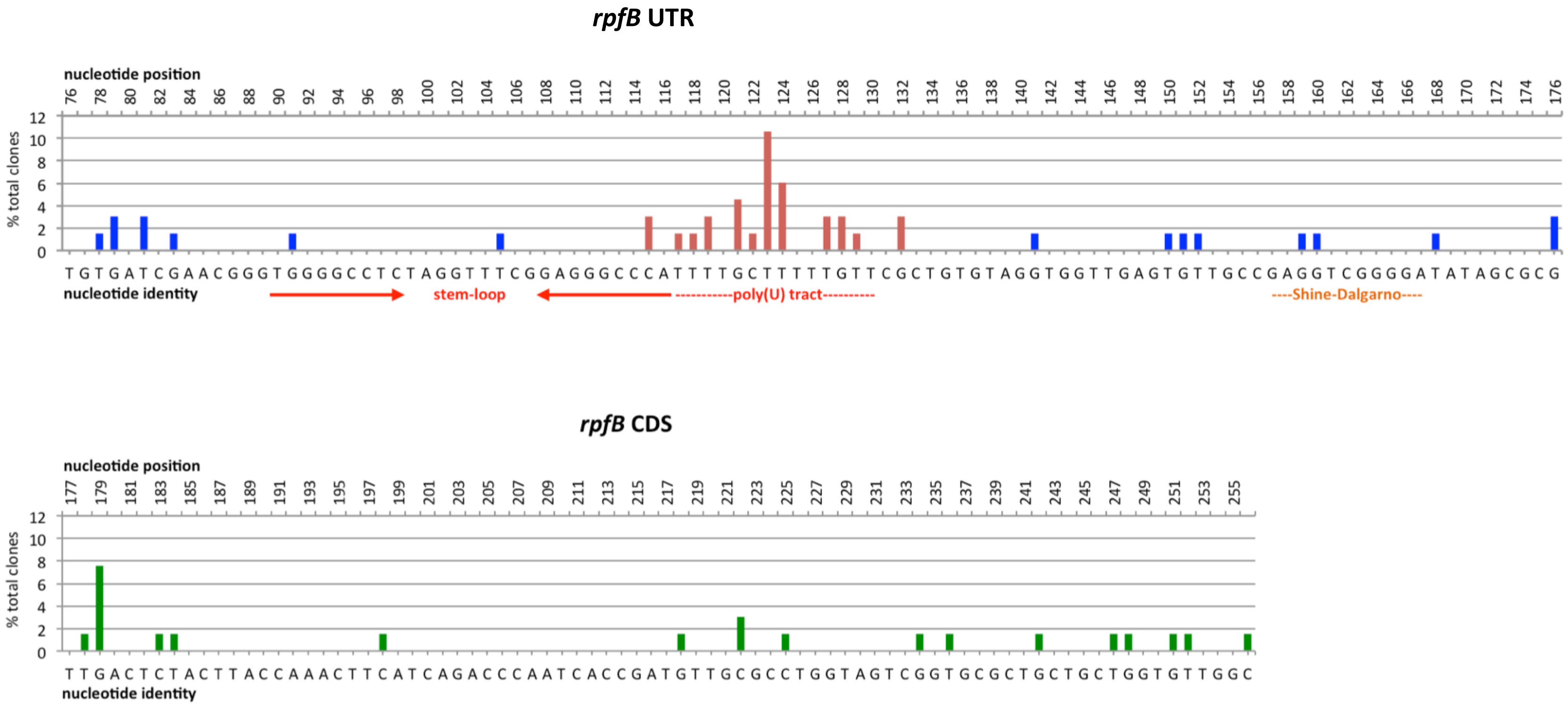

Fig. S3: Details of 3’ RACE. The figure illustrates the frequency of each 3’ terminus of a total of 66. Top panel shows positions within the 5’ UTR; bottom panel, positions within the coding region, according to the new TTG translation start site. The terminator (stem-loop and poly-U tract) as well as the presumed SD sequence have been indicated.

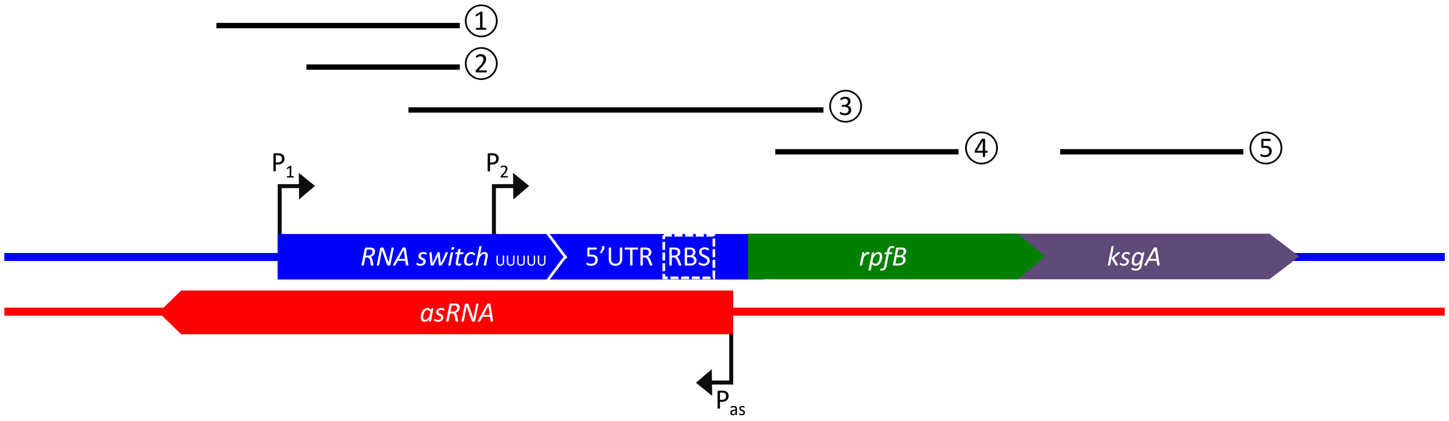

amplicon	target	forward primer	reverse primer
① 107 bp	asRNA	8.58, upstream of TSS_1	8.52, within 5' UTR
② 72 bp	5' UTR	8.11, upstream of TSS_2	8.52, within 5' UTR
③ 332 bp	P_1 *rpfB* readthrough	8.56, upstream of TSS_2	8.57, within *rpfB* CDS
④ 235 bp	*rpfB* from P_1 or P_2	8.07, within *rpfB* CDS	8.08, within *rpfB* CDS
⑤ 231 bp	*ksgA* from P_1 or P_2	8.09, within *ksgA* CDS	8.10, within *ksgA* CDS

Fig. S4: Outline of the *rpfB* locus and individual cDNA amplicons, shown as black lines above the genes. Primers for amplicons are shown in the table. The values for P_1 *rpfB readthrough as a percentage of 5' UTR = ③ / ② × 100*

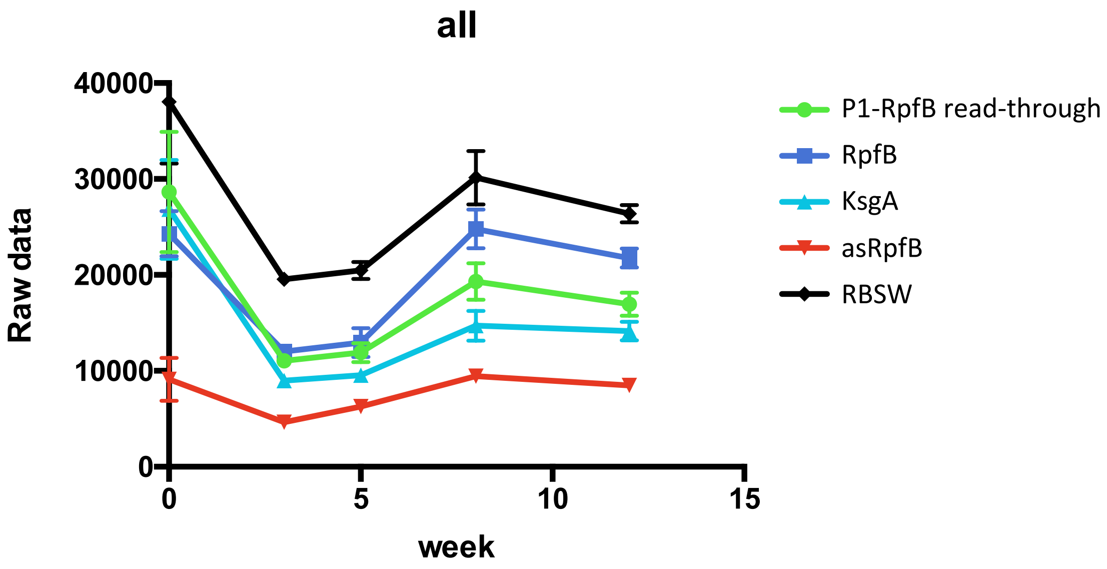

Fig. S5: Raw values for individual transcripts from the *rpfB* locus as determined by the amplicons indicated in Fig. S4. Values represent the mean and SD of three biological replicates.

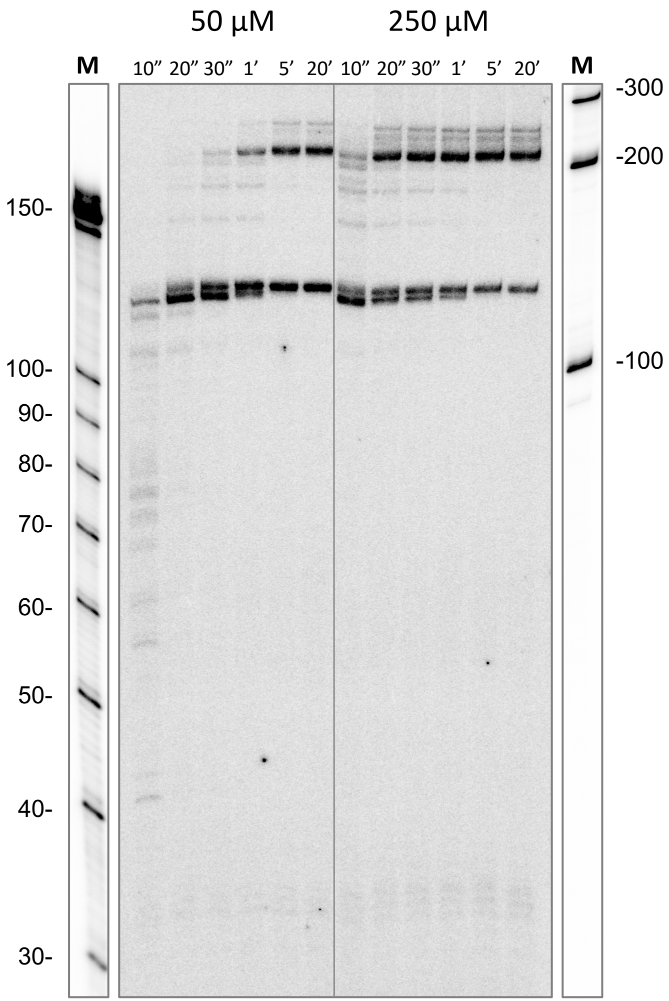

Fig. S6: Reducing elongation rate of RNA polymerase by lowering the NTP concentration. Single-round *in vitro* transcription of the RpfB 5' UTR using *E. coli* RNAP was carried out with 50 μM or 250 μM NTPs. Initiation complexes were stalled at position 11 by omitting UTP and elongated in the presence of heparin and 50 μM NTP (left gel) or 250 μM NTP (right gel).

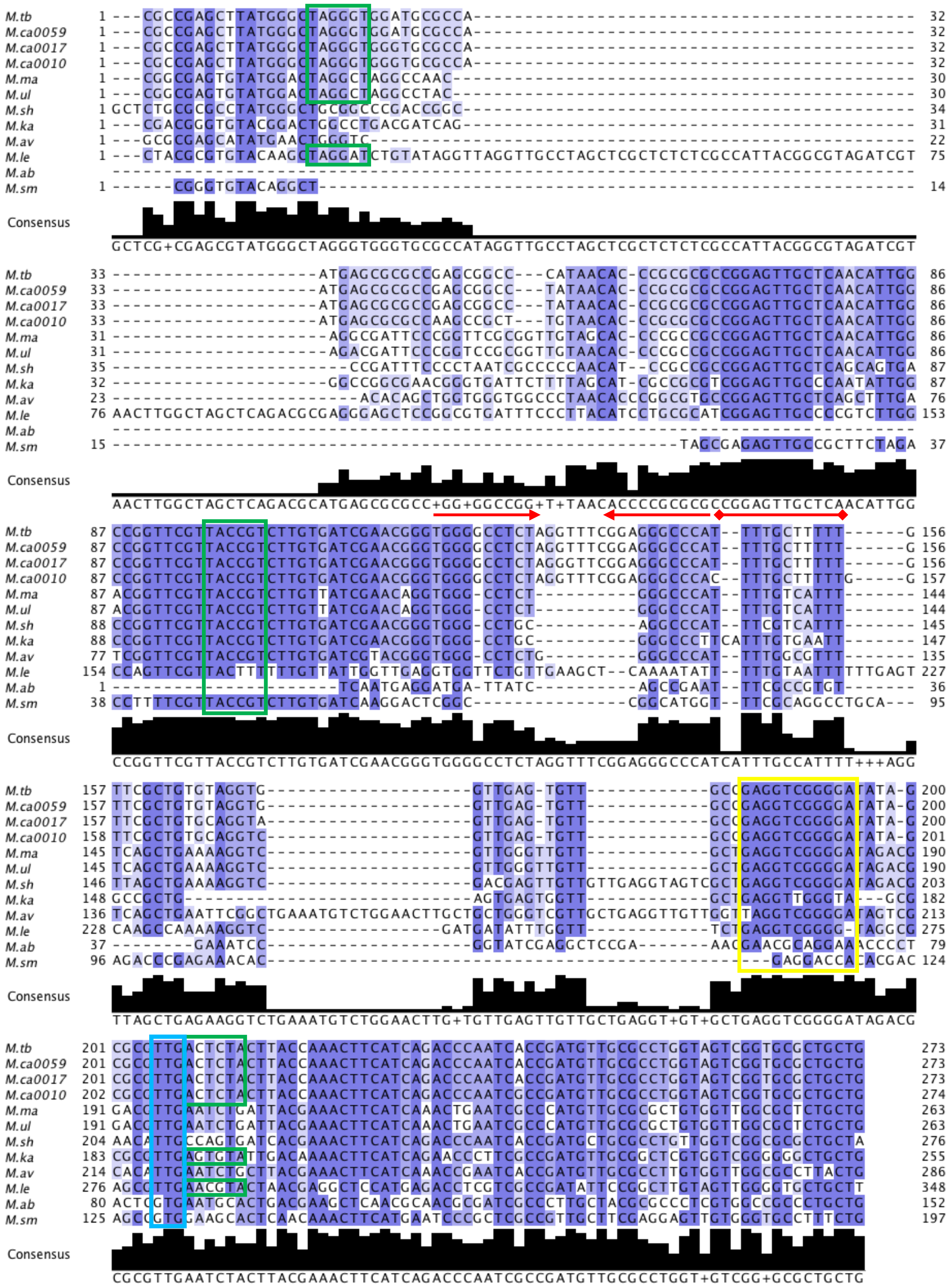

Fig. S7: Extended alignment of *rpfB* promoter regions and 5' UTRs. Additional species are: *M. leprae* (*M.le*), *M. abscessus* (*M.ab*), *M. smegmatis* (*M.sm*). Key is the same as in Fig. 9.
